# Roles of intralesional bacteria in the initiation and progression of oral squamous cell carcinoma

**DOI:** 10.1002/cam4.70209

**Published:** 2024-09-20

**Authors:** Zhuoyan Luo, Shiping Lv, Fangzhi Lou, Li Yan, Jingyi Xu, Ning Kang, Yunmei Dong, Xin Jin

**Affiliations:** ^1^ College of Stomatology Chongqing Medical University Chongqing China; ^2^ Chongqing Key Laboratory of Oral Diseases Chongqing China; ^3^ College of Medical Informatics Chongqing Medical University Chongqing China

**Keywords:** intralesional bacteria, microenvironment, oral microbiota, oral potentially malignant disorders, oral squamous cell carcinoma

## Abstract

**Background:**

Oral squamous cell carcinoma (OSCC) is the predominant form of head and neck cancer, often diagnosed at late stages, resulting in a poor prognosis. Recent studies indicate a potential association between OSCC and microbial presence. Microorganisms have been identified in various tumors and lesions, including OSCC and oral potentially malignant disorders (OPMDs). Intralesional microbiota are considered important components of the tumor microenvironment (TME) and may contribute to carcinogenesis.

**Methods:**

Sources were collected through thorough searches of databases PubMed and Embase. The review focused on microbial characteristics, potential origins, and their impact on cancer progression.

**Results:**

Bacteria display varying abundance and diversity throughout the stages of OSCC and OPMDs. Intraleisional bacteria may have diverse sources, including not only oral plaque and saliva but also potentially the gut. Intralesional bacteria have both pro‐carcinogenic and anti‐carcinogenic effects, affecting processes like cell proliferation, invasion, and immune response.

**Conclusions:**

Intralesional microbiota are crucial in OSCC and OPMDs, influencing both disease progression and treatments. Despite their significance, challenges like inconsistent sampling and microbial identification remain. Future research is required to fully understand their role and improve clinical applications.

## 1 INTRODUCTION

Oral squamous cell carcinoma (OSCC) is the most prevalent head and neck malignancy worldwide, comprising approximately 90% of oral malignant tumors.^1^ OSCC is characterized by a poor prognosis, primarily due to the inconspicuous early symptoms and high metastatic potential.^2^ OSCC often initiates from oral potentially malignant disorders (OPMDs), such as oral lichen planus (OLP), oral leukoplakia (OLK), and proliferative verrucous leukoplakia (PVL).^3^ The majority of OSCC cases are correlated with risk factors such as smoking, alcohol consumption, and betel nut chewing.^4^, ^5^ However, the etiology of OSCC remains incompletely understood. In recent years, numerous studies have suggested the association between microbiota and oral cancer.

The human microbiota is a comprehensive term that includes all microorganisms found on and within the human body.^6^ Imbalances of the human microbiota can cause or exacerbate various diseases, such as inflammatory bowel disease (IBD), autoimmune disorders, and even cancer.^7^, ^8^ Cancer arises from a combination of genetic and environmental factors, and approximately one‐fifth of which can be associated with the human microbiome.^9^ In contrast to the oral and gut microbiota, intralesional microbiota has received less attention in the past due to its minimal biomass.^10^ In recent years, the rapid development of sequencing technology has brought intralesional microbiota under the spotlight.^11^ Microbiota have been extensively confirmed to exist in various types of tumors, with distinct microbial compositions.^12^ Despite the low biomass, intralesional microbiota play a crucial role in cancer development through various mechanisms, including promoting proliferation and metastasis, inducing immunosuppression, and altering cell metabolism.^10^, ^13^, ^14^, ^15^

Oral cavity is the second‐largest microbiota in the body after the gut, with over 700 identified bacterial species.^16^ Emerging evidence has verified the existence of intralesional microbiota in OPMDs and OSCC, suggesting a potential impact on these disorders.^17^, ^18^ However, previous reviews on microbiota and OSCC mostly relied on surface samples, such as saliva and swabs. Microbial communities in surface samples are unstable and influenced by factors like oral hygiene, tobacco use, and diet.^19^ It is important to note that there are significant differences in microbial compositions and community structures between surface and tissue samples.^20^, ^21^ According to reports, bacteria constitute the majority of the intralesional microbiota, while fungi and viruses constitute a smaller proportion.^18^, ^22^ Therefore, this review aims to summarize the characteristics of intralesional bacteria, including representative bacterial alterations in OPMDs and OSCC, the possible origins of these bacteria, and their potential roles and mechanisms in the initiation and progression of OSCC.

## 2 DYNAMIC ALTERATIONS OF INTRALESIONAL BACTERIA IN OPMDs AND OSCC

There is a significant correlation between bacterial community structure and the progression of diseases. Diversity variations reflect overall bacterial changes. While conclusions of the studies are not entirely consistent, multiple results indicated that the bacterial diversity in OPMDs lesions often increases or remains unchanged, whereas a significant decrease is observed in OSCC compared to the control groups.^18^, ^23^, ^24^, ^25^, ^26^ Particularly, studies indicated the decrease of bacterial diversity during the progression from PVL to OSCC, and the significant loss of diversity in advanced T4 stage OSCC compared to the early stage.^18^, ^27^, ^28^ Changes in biodiversity may be attributed to the increased growth of potential pathogens during disease progression. Additionally, to clarify the specific bacterial compositions within OPMDs and OSCC lesions, the 16S rRNA gene sequencing results are summarized, as shown in Table 1. The microbial composition within the tissue is intricately related to disease subtypes, lesion sites, patient age, and other factors.^12^, ^29^, ^30^, ^31^

**TABLE 1:** Characteristics of the included studies.

Study, year	Participants (*N*)	Average/range of age (years)	Group	Bacterial ateration (increase, decrease)
Herreros‐Pomares et al., 2023^18^	Homogenous OLK (9) PVL (12) OSCC (10) PVL‐OSCC (8) HCs (11)	‐	Homogenous OLK vs. HCs	**Species**: *Streptococcus parasanguinis, Streptococcus salivarius, Fusobacterium periodonticum, Prevotella histicola, Porphyromonas pasteri, Megasphaera micronuciformis*
			PVL vs. HCs	**Phylum**: *Campilobacterota, Proteobacteria*
				**Genus**: *Granulicatella, Gemella, Eubacterium, Actinomyces, Deep Sea Euryarchaeotic Group* (*DSEG*)
				**Species**: *Prevotella salivae, Campylobacter concisus, Dialister pneumosintes, Schaalia odontolytica*
			PVL‐OSCC vs. HCs	**Phylum**: *Fusobacteriota, Campilobacterota, Proteobacteria*
				**Species**: *Lachnospiraceae bacterium, Selenomonas sputigena Prevotella shahii*
			OSCC vs. HCs	**Phylum**: *Fusobacteriota*
				**Species**: *Capnocytophaga leadbetteri, Capnocytophaga sputigena, Capnocytophaga gingivalis, Campylobacter showae, Metamycoplasma salivarium, Prevotella nanceiensis*
Khan et al., 2023^26^	OSCC (9) OPMDs(39) HCs (18)	68 (49–80) 67 (30–81) 66 (44–92) 66 (51–73)	OSCC/OPMDs vs. HCs	**Genus**: *Fusobacterium, Shewanella*
Pratap Singh et al., 2023^28^	OSCC (60) OPMDs (15) NPT (20)	‐	OSCC vs. OPMDs	**Phylum**: *GNO2, Verrucomicrobia, TM7, Spirochaetes, Bacteroidetes, Firmicutes, Actinpbacteria* **Genus**: *Peptostreptococcaceae, Comamonadaceae, Porpliyromonas, Prevotella, Pseudomonas, Treponema, Lachnospiraceae, Leptotrichla, Capnocytophaga, Desulfovibricnaceae, Oscillospira, Streptococcus, Rothia, Arcanobacterium, Clostridiales, Parvimonas* **Species**: *Capnocytophaga, Streptococcus, Rothia*
			OSCC vs. NPT	**Phylum**: *Proteobacteria* **Species**: *Rothia mucilaginosa, Rothia dentocariosa, Neisseria mucosa*
			OPMDs vs. NPT	**Phylum**: *Spirochetes, Bacteroidetes, TM7*
Herreros‐Pomares et al., 2021^27^	PVL (10) HCs (5)	60–86 ‐	PVL vs. HCs	**Genus**: *Mobiluncus, Peptoniphilus, Anaerosalibacter, Stomatobaculum, Pacearchaeota Incertae Sedis AR13, Bacteroides, Johnsonella, Cellulosibacter, Terrimicrobium, Acetobacteroides, Phocaeicola*
				**Species**: *Oribacterium* sp. *oral taxon 108, Campylobacter jejuni, uncultured Eubacterium* sp.*, Campylobacter, Tannerella, Porphyromonas, Peptoniphilus, undefined Mobiluncus, Anaerosalibacter bacteria, Streptococcus agalactiae, Mycoplasma adleri (T), uncultured Fusobacteria, Bacteroidales, Synergistetes, Mycoplasma, Bacteroides, undefined Acetobacteroides, Olsenella, Thermoflavifilum, Cellulosibacter, Pacearchaeota Incertae Sedis AR13*
Wang et al., 2020^19^	Reticular OLP (12) Erosive OLP (12) HCs (8)	47.429 ± 10.799 36.125 ± 14.126 38.500 ± 13.554	OLP vs. HCs	**Genus**: *Escherichia–Shigella, Phyllobacterium, Megasphaera, Bacteroides, Streptococcus, Micrococcus, Sphingobium*
				**Species**: *Methylobacterium aquaticum, Achromobacter xylosoxidanssubsp. Xylosoxidans, Sphingomonas mali, Pseudomonaspoae, Tsukamurella tyrosinosolvens, Rhodopseudomonas pentothenatexigens, Megasphaera elsdenii DSM 20460*
Decsi et al., 2019^25^	OPMDs (7) NPT (7)	54–85	OPMDs vs. NPT	**Species**: *Fusobacterium nucleatum, Streptococcus oralis, Streptococcus mitis, Streptococcus pneumoniae, Gemella haemolysans*
Choi et al., 2016^32^	OLP (36) HCs (10)	‐	OLP vs. HCs	**Phylum**: *Bacteroidetes, Firmicutes* **Genus**: *Streptococcus* **Species**: *Fusobacterium nucleatum, Neisseria oralis, C. gingivalis, Leptotrichia hongkongensis, Eikenella corrodens, T. denticola, T. socranskii, Centipeda periodontii, Selenomonas sputigena*
He et al., 2023^33^	OSCC (11) NPT (11)	49 ± 12	OSCC vs. NPT	**Phylum**: *Fusobacteriota, Spirochaetota, Bacteroidota, Campylobacterota, Proteobacteria, Actinobacteriota*
				**Genus**: *Fusobacterium, Campylobacter, Prevotella, Ralstonia, Staphylococcus*
				**Species**: *Fusobacterium nucleatum*
Nie et al., 2022^35^	OSCC (37) NPT (37)	57.9 ± 9.6 58.1 ± 9.7	OSCC vs. NPT	**Phylum**: *Firmicutes, Bacteroidetes, Fusobacteria, Spirochaetes, Proteobacteria* **Genus**: *Fusobacterium, Prevotella, Porphyromonas, Campylobacter, Aggregatibacter, Treponema, Peptostreptococcus, Stenotrophomonas, Neisseria, Sphingomonas, Veillonella* **Species**: *Porphyromonas endodontalis, Campylobacter gracilis, Peptostreptococcus stomatis, prevotella intermedia, Eubacterium vuril subsp schtika, Parvimonas micra, Pseudomonas beteli, Rothia mucilaginosa, Sphingomonas alpina, Veillonella dispar*
Michikawa et al., 2022^38^	OSCC (33) NPT (36)	‐	OSCC vs. NPT	**Genus**: *Fusobacterium, Rothia, Streptococcus*
Zeng et al., 2022^40^	OSCC outer tissues (22) OSCC inner tissues (11) NPT(16)	‐	OSCC outer tissues vs. NPT	**Genus**: Fusobacterium, Prevotella, Porphyromonas, *Streptococcus, Capnocytophaga, Neisseria, Prevotella, Veillonella, Alloprevotella*
			OSCC outer tissues vs. OSCC inner tissues	**Genus**: *Fusobacterium, Neisseria, Porphyromonas, Alloprevotella, Prevotella, Selenomonas, Parvimonas*
Zhang et al., 2022^30^	Young OSCC (20) Elder OSCC (20)	37.55 (26–46) 65.6 (59–80)	Younger OSCC vs. Elder OSCC	**Genus**: *Ochrobactrum, Prevotella, Ralstonia, Pedobacter*
Li et al., 2022^34^	OSCC(132) HCs(20)	60.52 ± 13.14 58.31 ± 12.72	OSCC vs. HCs	**Phylum**: *Fusobacteria, Spirochaetes, Firmicutes, Actinobacteria*
				**Genus**: *Fusobacterium, Treponema, Capnocytophaga*
Gopinath et al., 2021^20^	OSCC (48) HCs (46)	49.31 ± 13.24 50.67 ± 6.81	OSCC vs. HCs	**Genus**: *Solobacteria, Peptostreptococcus, Catonella, Finegoldia, Campylobacter, Prevotella, Capnocytophaga, Corynebacterium, Actinomyces, Rothia, Streptococcus*
Sarkar et al., 2021^23^	OSCC (50) NPT (50)	‐	OSCC vs. NPT	**Phylum**: *Bacteroidetes, Deinococcus, Proteobacteria, Firmicutes, Actinobacteria* **Genus**: *Prevotella, Corynebacterium, Pseudomonus, Deinococcus, Noviherbaspirillum, Serratia, Anoxybacilus, Stenotrophomonas, Sutterella, Actinomyces, Bacillus, Lysobacter, Paenibacillus, Ammoniphilus, Bifidobacterium, Megamonas, Collinsella, Brevibacillus, Megasphaera, Blautia, Methylobacterium, Roseburia, Phenylobacterium, Pseudopropionibacterium, Parabacteroides, Anaerobacillus*
				**Species**: *Capnocytophaga* sp.*, unidentified Micrococcaceae, uncultured Cornebacterium 1, Roseburia* sp.*, Uncultured Sphingomonas, Unidentified Bacillaceae, Phenylobacterium* sp.*, Uncultured Roseburia, Methylobacterium* sp.*, Uncultured Megasphaera, Uncuiltured Pseudopropionibacterium, Uncultured Abiotrophia, Uncultured Anoxybacillus, Blautia* sp.*, Uncultured Blautia, Uncultured Prevotella 9, Messilia* sp.*, Brevibacillus* sp.
Yang et al., 2021^21^	OSCC (23) NPT (23)	61.9 ± 12.3	OSCC vs. NPT	**Phylum**: *Campylobacterota, Actinobacteria*
				**Genus**: *Campylobacter, Gemella, Filifactor, Catonella, Veillonella, Granulicatella*
				**Species**: *Aggregatibacter segnis, Campylobacter showae, Gemella, Morbillorum, Rothia mucilaginosa, Granulicatella adiacens, Streptococcus sanguinis, Veillonella rogosae*
Ye et al., 2021^36^	OSCC (tougue) (23) NPT (23)	63.0 ± 9.6	OSCC (tougue) vs. NPT	**Phylum**: *Bacteroidetes, Spirochaetes, Actinobacteria*
				**Genus**: *Rothia, Neisseria, Streptococcus, Actinomyces*
				**Species**: *Streptococcus pneumoniae*
Torralba et al., 2021^39^	OSCC (18) NPT (18)	‐		**Genus** *:Fusobacterium, Parvimonas, Peptostreptococcus, Porphyromonas, Prevotella, Streptococcus, Veillonella*
Li et al., 2020^24^	OSCC (gingiva) (10) NPT (10)	61 ± 9.49	OSCC (gingiva) vs. NPT	**Phylum**: *Proteobacteria*
				**Genus**: *Aggregatibacter, Rothia, Granulicatella, Lactobacillus*
Zhou et al., 2020^37^	OSCC (24) NPT (24)	57.2	OSCC vs. NPT	**Phylum**: *Bacteroidetes, Firmicutes, Fusobacteria*
				**Genus** *:Fusobacterium, Treponema, Streptococcus, Peptostreptococcus, Carnobacterium, Parvimonas, Tannerella, Filifactor, Streptophyta, Brevundimonas, Paenibaillus, Microbacterium, Desulfovibrio, Mucispirillum, Arthrobacter*
Chang et al., 2019^41^	OSCC (61) NPT(61) HCs (30)	57.4 ± 10.4 57.4 ± 10.4 55.4 ± 10.2	OSCC vs. HCs	**Genus** *:Fusobacterium nucleatum, Porphyromonas gingivalis, Streptococcus sanguinis*
Zhang et al., 2019^42^	OSCC (50) NPT (50)	61	OSCC vs. NPT	**Genus**: *Fusobacterium, Alloprevotella, Leptotrichia, Porphyromonas, Capnocytophaga, Aggregatibacter, Campylobacter, Selenomonas, Treponema, Catonella, Filifactor, Parvimonas, Peptococcus, Peptostreptococcaceae incertae sedis, Streptococcus, Veillonella, Rothia, Haemophilus, Neisseria, Lautropia, Actinomyces, Granulicatella, Corynebacterium, Granulicatella, Oribacterium, Tannerella*
				**Species**: *Fusobacterium nucleatum, Prevotella intermedia, Aggregatibacter segnis, Capnocytophaga leadbetteri, Peptostreptococcus stomatis, Porphyromonas catoniae, Catonella morbi, Campylobacter rectus, Aggregatibacter segnis, Gemella morbillorum, Streptococcus oralis, Granulicatella elegans, Granulicatella adiacens*
Perera et al., 2018^4^	OSCC (25) FEP (27)	‐	OSCC vs. FEP	**Genus**: *Atopobium, Pseudomonas, Capnocytophaga, Lautropia, Staphylococcus, Propionibacterium, Sphingomonas, Delftia, Agrobacterium, Afipia, Burkholderiales G, Mycobacterium, Flavitalea, Diaphorobacter, Stenotrophomonas, Schlegelella, Bosea, Pedobacter, Acinetobacter, Yersinia, Methylobacterium, Cardiobacterium, Achromobacter, Ralstonia, Corynebacterium*
				**Species**: *Fusobacterium sp HOT 204, Prevotellaloeschei, Prevotella salivae, Campylobacter concisus, Streptococcus mitis, Streptococcus sp oraltaxon, 070, Lautropia mirabilis, Rothia dentocariosa, Leptotrichiasp oral taxon 225, Propionibacterium acnes, Actinomyces massiliensis, Rothia aeria, Streptococcus sanguinis, Streptococcus dentisani, Streptococcus sp oral taxon 058, Streptococcus australis, Delftia acidovorans, Burkholderiales G sp 0ral TaxonA57, Haemophilus parahaermolyticus, Streptococcus ristatus, Afipia broomeae, Favitalea sp oral taxon 320 nov 8710*
Hooper et al., 2006^31^	Superficial OSCC (20) Deep OSCC (12) NPT (19)	66.9 ± 12.7	OSCC vs. NPT	**Species**: *Exiguobacterium oxidotolerans, Prevotella melaninogenica, Staphylococcus aureus, Veillonella parvula, Micrococcus luteus, Fusobacterium naviforme, Prevotella* sp. (*oral clone BE073 phylotype*)*, Rothia mucilaginosa, Streptococcus salivarius, Actinomyces odontolyticus, Moraxella osloensis, Prevotella veroralis, Propionibacterium acnes, Atopobium parvulum, Streptococcus parasanguinis, Veillonella dispar, Streptococcus mitis/oralis*

Abbreviations: ‐, not mentioned; deep OSCC, consisting entirely of tissue from within the tumor mass; FEP, fibroepithelial polyp; HCs, healthy controls; NPT, normal paralesional tissues; OLK, oral leukoplakia; OLP, oral lichen planus; OPMDs, oral potentially malignant disorders; OSCC inner tissues, collected at the front of the invasion of the tumor; OSCC outer tissues, collected at a depth of less than 2 mm on the surface of the tumor; OSCC, oral squamous cell carcinoma; PVL, proliferative verrucous leukoplakia; PVL‐OSCC, OSCC preceded by the evolution of PVL; superficial OSCC, consisting of tissue from within and from the surface of the tumor.

Researches regarding the bacterial compositions of OPMDs tissues are still lacking. A study involving various OPMDs revealed a significant increase in the relative abundance of *Fusobacterium nucleatum* (*F. nucleatum*) and a decrease in *Streptococcus* species, compared to the healthy mucosa.^25^ PVL is considered a severe subtype of OLK and exhibits the highest malignant transformation rate among OPMDs.^3^ PVL showed a loss of diversity and enrichment of pathogens such as *Oribacterium* sp. *oral taxon 108* and *Campylobacter jejuni*.^27^ As PVL progressed to OSCC, the enrichment of *Lachnospiraceae, Selenomonas sputigena*, etc. was observed.^18^ Additionally, another study indicated the depletion of *Streptococcus* and *Rothia* during the oral carinogenesis of OPMDs.^28^

**FIGURE 1:**
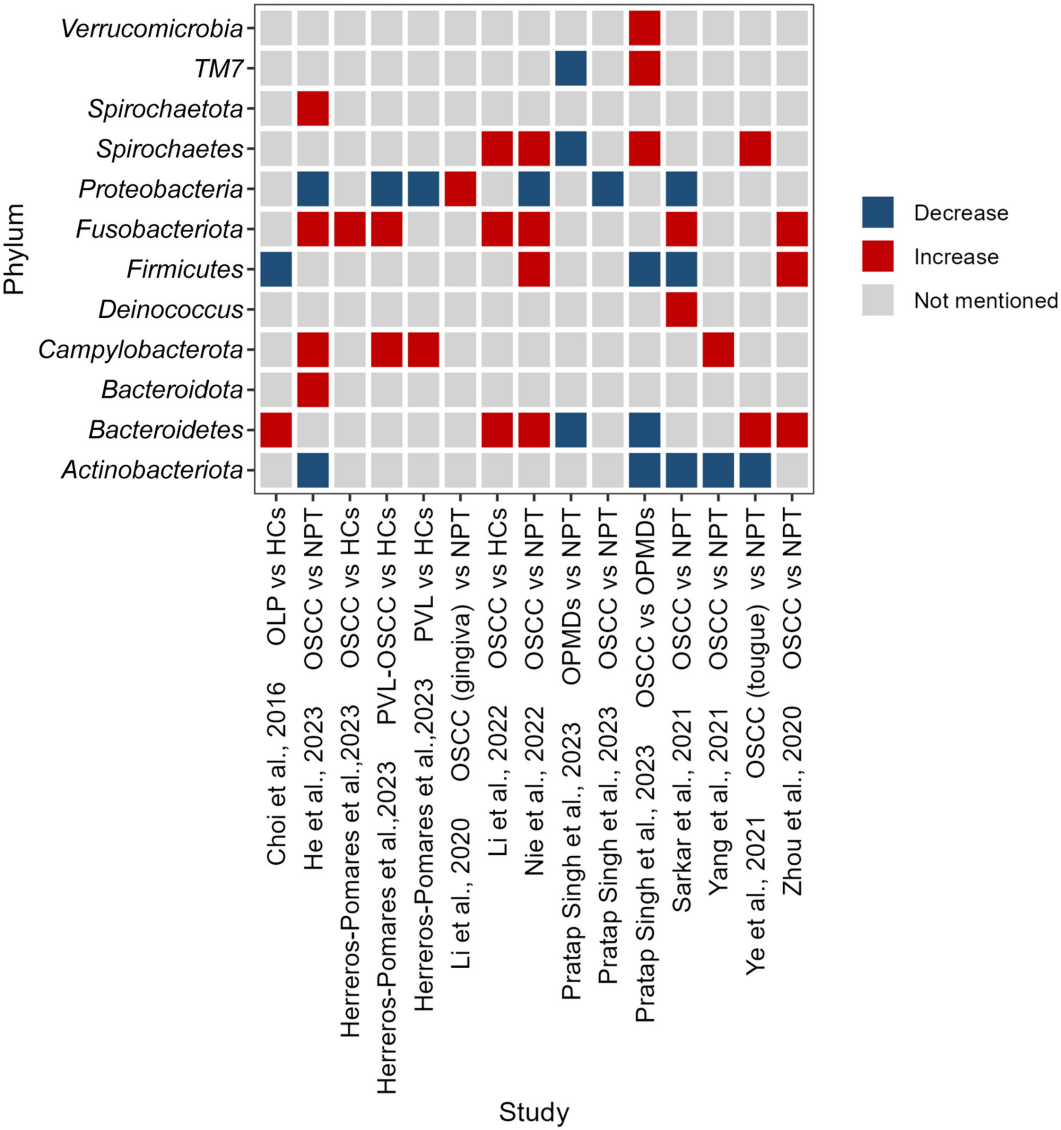
The reported trend of abundance changes of bacteria in OSCC or OPMDs at the phylum level.

**FIGURE 2:**
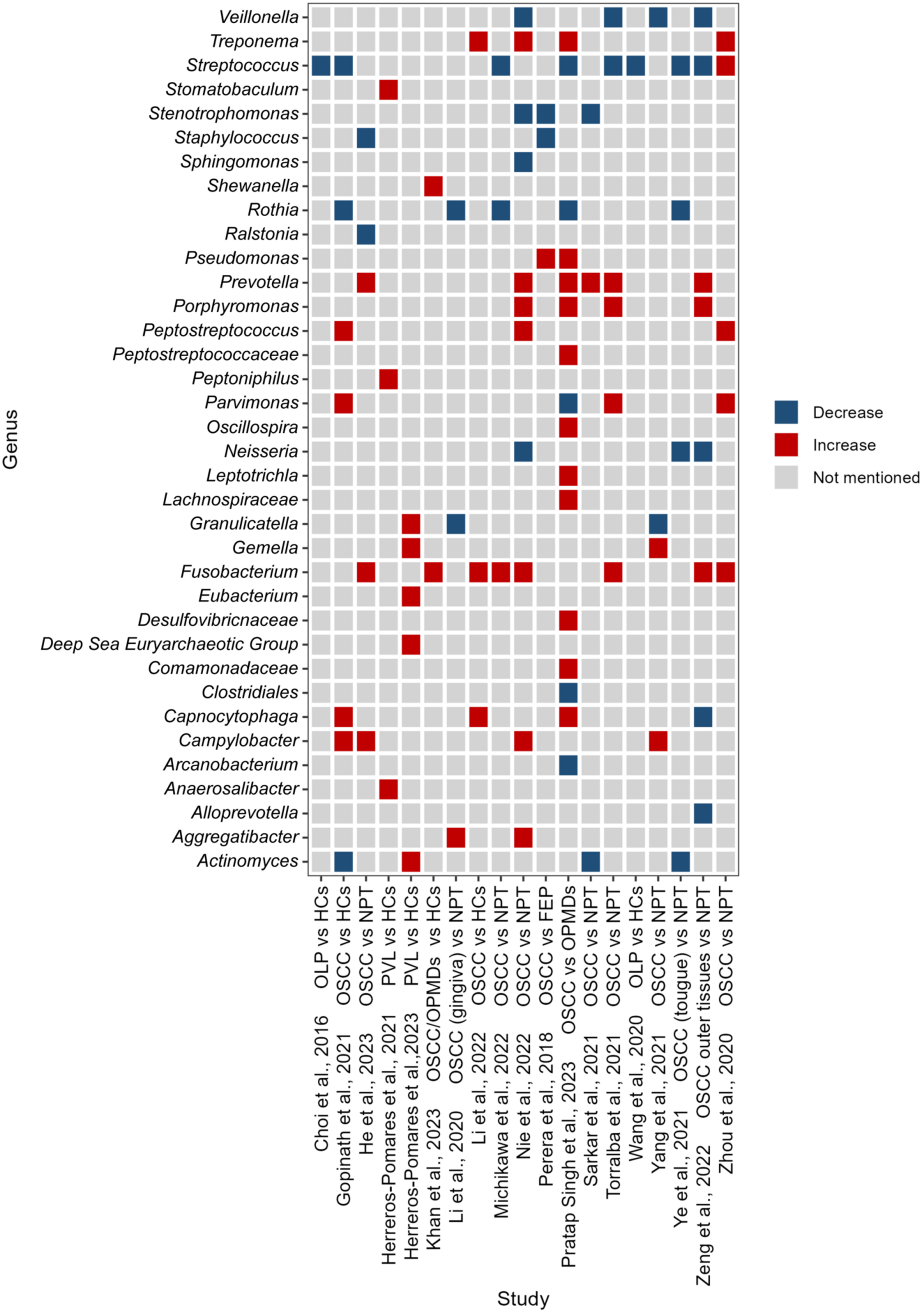
The reported trend of abundance changes of bacteria in OSCC or OPMDs at the genus level.

The trends of commonly identified bacteria in OSCC are analyzed using a heatmap, as shown in Figures 1 and 2. At the phylum level, the abundances of *Fusobacteria* and *Campylobacterota* significantly increased, whereas *Actinobacteriota* significantly decreased. However, the reported trends of changes in 4 phyla in OSCC lesions show inconsistencies^18^, ^21^, ^23^, ^24^, ^28^, ^32^, ^33^, ^34^, ^35^, ^36^, ^37^ (Figure 1). At the genus level, 8 genera, including *Fusobacterium*, significantly increased in OSCC, while 5 genera including *Veillonella* and *Stenotrophomonas* significantly decreased. Changes of 5 genera show inconsistencies in OSCC^4^, ^18^, ^19^, ^20^, ^21^, ^23^, ^26^, ^27^, ^28^, ^32^, ^33^, ^34^, ^35^, ^36^, ^37^, ^38^, ^39^, ^40^ (Figure 2). Specially, a higher level of *F. nucleatum* in OSCC has been widely reported.^25^, ^33^, ^41^, ^42^, ^43^ Moreover, bacteria show specific compositions across different stages of OSCC. In the early stages, genera *Clostridiales*, *Desulfovibrionaceae*, and *Leptotrichia* are enriched, while *Pedobacter* is enriched in the late stage.^28^ From the early to late stage, the relative abundance of *Solobacterium moorei* is positively correlated with OSCC progression.^21^ The prevalence of *Porphyromonas gingivalis* (*P. gingivalis*) was found to be elevated in patients with T3‐T4 stage OSCC, poorly differentiated subtypes, and lymph node metastasis.^41^ In addition, phyla *Firmicutes*, *Actinobacteria*, and *Fusobacteria*‐related taxa exhibit notable predictive accuracy in diagnosing or prognosing OSCC.^34^ Increased levels of *Capnocytophaga* and reduced levels of *Streptococcus* could potentially act as biomarkers for advanced OSCC.^28^

Traditional 16S rRNA gene sequencing is limited to genus‐level microbial community classification and does not support accurate species‐level identification. Future studies may leverage more advanced sequencing methods like 5R 16S rRNA gene sequencing and 2bRAD sequencing to explore the microbial composition in tissues with low microbial load, enabling precise genus‐level results in the investigation of OPMDs and OSCC lesions.^12^, ^44^

## 3 POTENTIAL ORIGINS OF THE INTRALESIONAL MICROBIOTA

The current research has mentioned the potential origins of intralesional microbiota, particularly emphasizing the association with periodontitis. Periodontal pathogens, especially *P. gingivalis*, dominate the entire periodontitis‐associated OSCC process.^29^ Periodontal pathogens are enriched in cancerous tissue, with bacterial distributions similar to those in subgingival plaque. The relative abundance of the key components, *F. nucleatum* and *P. gingivalis*, is significantly positively correlated with that in subgingival plaque.^41^ *Streptococcus anginosus* (*S. anginosus*) shares the same genotype in OSCC tissue and dental plaque.^45^ Another research revealed identical *P. gingivalis* nucleotide sequences in OSCC tissue and saliva, along with the consistent distribution of *fimA* genotypes in both, suggesting that *P. gingivalis* in OSCC may originate from the salivary microbial reservoir.^46^ Microbial compositions in the lesions are similar to those in adjacent normal tissues, which suggest that intralesional bacteria might originate from nearby tissues.^35^, ^41^ In OLP, chronic inflammation may alter epithelial permeability and disrupt the epithelial barrier, allowing bacteria to penetrate the epithelial and lamina propria.^19^ Specifically, certain gut‐specific bacteria were also detected in OSCC, such as *Clostridium neonatale*.^41^ This indicates intraleisional bacteria may have sources other than the oral cavity, such as the intestines or other anatomical sites.

With the knowledge of the intralesional microbiotas and related studies, it can be inferred that microorganisms within OSCC and OPMDs tissues have multiple sources. Revealing the sources of intralesional microbiota helps understand the mechanisms involved in OPMDs and OSCC, and provides inspiration for disease prevention and treatment.

## 4 POTENTIAL ROLES OF INTRALESIONAL BACTERIA IN OSCC INITIATION AND PROGRESSION

Polymorphic microbes have been considered an emerging hallmark of cancer.^47^ As a crucial component of the tumor microenvironment (TME), microbiotas play complex roles in cancer development. Intralesional bacteria can contribute to carcinogenesis of normal cells or exacerbate existing OSCC via various mechanisms, including promoting cell proliferation, inhibiting apoptosis, and inducing chronic inflammation and immune suppression.

### 4.1 Promote cell proliferation

Continuous cell proliferation is a critical characteristic of cancer. Normal cells maintain cellular homeostasis by finely regulating growth signals.^48^ However, the presence of intralesional bacteria disturbs this balance and accelerates the proliferation of epithelial cells or cancer cells.^49^

Proteomic analyses have revealed that *P. gingivalis* leads to a significant upregulation in the levels of cyclins and cyclin‐dependent kinases (cdks) in oral epithelial cells, which accelerate transitions between different phases of the cell cycle.^50^, ^51^ Exposure to *P. gingivalis* or *F. nucleatum* activates toll‐like receptors (TLRs)/interleukin‐6 (IL‐6)/signal transducer and activator of transcription 3 (STAT3) axis in human oral keratinocytes (HOK), and induces cyclin D1 expression.^52^ In a specific environment containing ethanol, the interaction between *Streptococcus* and human papillomavirus (HPV) causes malignant phenotypes in HOK, including uncontrolled cell proliferation.^53^

**FIGURE 3:**
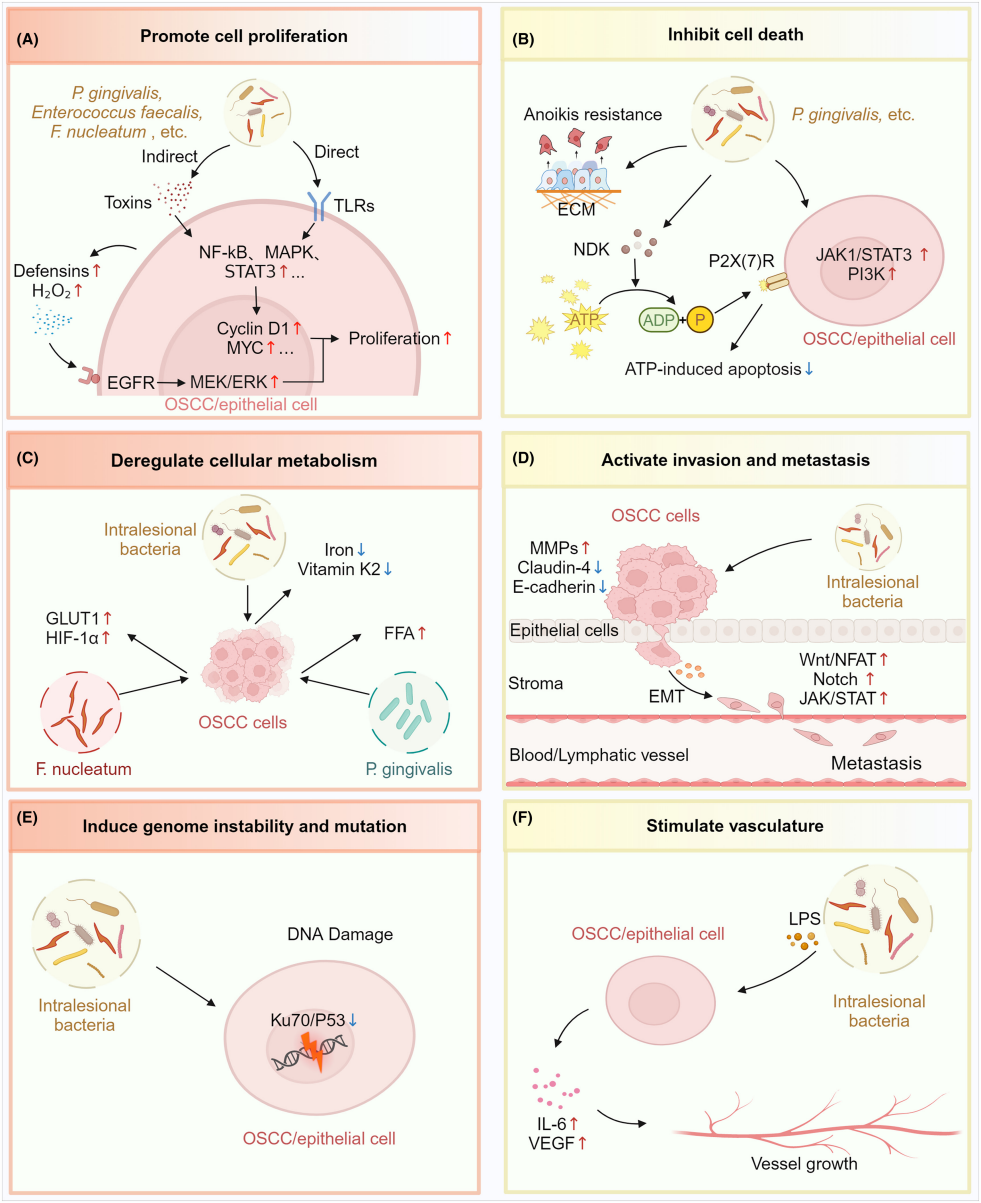
Mechanisms of intralesional microbiotas promoting the initiation and progression of OSCC. (A) Promoting cell proliferation. Intralesional bacteria, such as *P. gingivalis, F. nucleatum*, and *Enterococcus faecalis*, can directly promote cell proliferation or release toxins that facilitate this process. Through the activation of membrane receptors, such as toll‐like receptors (TLRs), the NF‐κB, MAPK, and STAT3 pathways are stimulated. The downstream cyclins and the MYC gene expression upregulate, which accelerates the cell cycle and promotes cell proliferation. The upregulation of human defensins triggered by *P. gingivalis* and the production of H_2_O_2_ triggered by *E. faecalis* can both activate Epidermal Growth Factor Receptor (EGFR), thereby promoting cell proliferation through pathways including MEK/ERK. (B) Inhibiting cell death. Microbiotas participate in inhibiting cell death through pathways such as JAK/STAT3 and PI3K. *P. gingivalis* ecto‐nucleoside diphosphate kinase (NDK) depletes extracellular ATP to reduce ATP‐dependent apoptosis mediated by P2X(7) receptor (P2X(7)R). *P. gingivalis* also induce resistance of host cells to anoikis, a process where loss of cell adhesion to the extracellular matrix (ECM) triggers cell death. (C) Deregulating cellular metabolism. Intralesional bacteria consume extracellular iron and vitamin K2, thus reducing the potential anticancer effects of these nutrients. Infection with *P. gingivalis* causes abnormal fatty acid metabolism in OSCC cells, resulting in elevated levels of free fatty acids (FFA). *F. nucleatum* upregulates the expression of glucose transporter 1 (GLUT1) and HIF‐1α in OSCC cells, which influences the metabolic processes involved in glucose metabolism and responses to hypoxia. (D) Activating invasion and metastasis. The increase in cytokines such as MMPs and the decrease in cell adhesion‐related proteins E‐cadherin and claudin‐4 promote loss of cell polarity and depleted cell–cell and cell‐ECM adhesion. Cytokines released by OSCC cells and epithelial cells facilitate OSCC EMT process through multiple pathways including Wnt/NFAT1, Notch, and JAK/STAT1. Subsequently, transformed cells are able to migrate through intercellular spaces, penetrate ECM, and infiltrate surrounding tissues or enter circulation to achieve distant metastasis (Adapted from “Blood Vessel (Straight, Light Background)”, by BioRender.com (2024). Retrieved from https://app.biorender.com/biorender‐templates). (E) Inducing genome instability and mutation. Intralesional bacteria cause DNA double‐strand breaks by suppressing the Ku70/P53 pathway. (F) Stimulating vasculature. Bacteria promote angiogenesis by stimulating vascular endothelial growth factor (VEGF) 2 production and promoting VEGF through the induction of IL‐6. (Image created with BioRender.com, with permission).

In OSCC, bacteria can relieve the restrictions on cell proliferation through multiple mechanisms. *P. gingivalis* accelerate G1 and S phases of cell cycle also via the upregulation of cyclin D1 through the miR‐21 (microRNA‐21)/PDCD4 (Programmed Cell Death Protein 4)/AP‐1 (Activator Protein‐1) negative feedback signaling pathway.^54^ Similarly, MYC is a crucial driver gene that accelerates the cell cycle. In OSCC, *Cutibacterium acnes* is strongly associated with the enriched MYC pathway.^55^ P53 is another crucial tumor suppressor protein that regulates cell cycle progression, DNA repair, and apoptosis. *F. nucleatum* enhances the proliferation ability of tongue squamous cell carcinoma cells by causing DNA damage via the downregulation of Ku70/P53 pathway.^56^ Epidermal growth factor receptor (EGFR) is a transmembrane tyrosine kinase receptor, which plays a crucial role in cellular processes including proliferation, migration and differentiation.^57^ Human defensins may serve as one of the ligands of EGFR. *P. gingivalis* infection upregulates human defensins expression and activates downstream EGFR signaling, thus promoting cell proliferation.^58^ *Enterococcus faecalis* produces hydrogen peroxide when interacting with host cancer cells. Hydrogen peroxide promotes EGFR activation, which contributes to proliferation potentially via mitogen‐activated protein kinase kinase/extracellular signal‐regulated kinase (MAP2K/ERK).^59^ *Treponema denticola* can invade Cal‐27 cells, directly promoting cell proliferation and inhibiting apoptosis via transforming growth factor‐beta (TGF‐β) pathway.^60^ Additionally, OSCC cells infected with bacteria show activated signaling pathways related to cell proliferation, including janus kinase 1/signal transducer and activator of transcription 3 (JAK1/STAT3), nuclear factor kappa B (NF‐κB), and phosphoinositide 3‐kinase (PI3K)^52^, ^61^, ^62^ (Figure 3A).

### 4.2 Inhibit cell death

Apoptosis is a form of programmed cell death, serving as a natural defense againist cancer progression. Evasion of cell death enables cells to prolong their survival and accumulate more mutations.^63^ Studies have shown that both epithelial cells and OSCC cells experience various anti‐apoptotic effects when co‐cultivated with bacteria.

*P. gingivalis* in gingival epithelial cells upregulates the expression of miR‐203, resulting in the reduction of downstream targets such as the suppressor of cytokine signaling 3 (SOCS3) mRNA. The decreased SOCS3 level downregulates apoptosis through JAK/STAT3 pathways.^64^ The B‐cell lymphoma‐2 (Bcl‐2) family comprises both anti‐apoptotic and pro‐apoptotic members, whose counterbalancing regulates the apoptotic trigger.^48^ A meta‐analysis revealed a significant upregulation of genes suppressing apoptosis in epithelial cells infected with *P. gingivalis*, including the anti‐apoptotic protein Bcl‐2.^65^ Disruption of normal adhesion between cells and the extracellular matrix (ECM) triggers another programmed cell death response called anoikis. *P. gingivalis* enables oral keratinocytes to develop anoikis resistance potentially through the Bcl‐2 protein.^66^ *P. gingivalis* ecto‐nucleoside diphosphate kinase (NDK) is an extracellular adenosine 5′‐triphosphate (ATP) enzyme. NDK can hydrolyse extracellular ATP, thereby inhibiting ATP‐dependent apoptosis mediated by purinergic receptor P2X(7).^67^ Furthermore, a separate study demonstrated that NDK inhibits apoptosis by phosphorylating Heat‐shock‐protein‐27 (Hsp27) in gingival epithelial cells. Phosphorylation of Hsp27 deactivates the proapoptotic Bcl‐2‐associated X protein (Bax) and suppresses the release of cytochrome c.^68^ *P. gingivalis* also inhibits cell apoptosis partly by controlling the intrinsic mitochondria cell death through a caspase‐dependent apoptotic pathway.^65^

In OSCC cells, studies revealed that the activation of TLR2 by bacteria leads to the upregulation of miR‐146a‐5p and subsequent suppression of caspase recruitment domain‐containing protein 10 (CARD10), which facilitates resistance to cell death.^69^ *T. denticola* inhibits apoptosis of cancer cells through TGF‐β pathway.^60^ *T. denticola* and its toxin *Treponema denticola* chymotrypsin‐like proteinase (Td‐CTLP) are significantly associated with higher expression of TLR7 and TLR9. The activation of mitogen‐activated protein kinase (MAPK) signaling via TLR7 and TLR9 significantly promotes cell survival^70^ (Figure 3B).

### 4.3 Deregulate cell metabolism

By disturbing cell metabolism, carcinogenic bacteria may create conditions conducive to the advancement of OSCC. NDK of *P. gingivalis* can hydrolyse extracellular ATP, which influences associated metabolic processes in oral epithelial cells.^67^ Stimulation of *P. gingivalis* W83 membrane components triggers a strong metabolic gene expression response in OSCC cells.^71^ Hypoxia‐inducible factor 1α (HIF‐1α) regulates cellular responses in low‐oxygen environments, affecting transcription, angiogenesis, and metabolism. OSCC cells show strong HIF‐1α expression in the cytoplasm, especially in areas densely populated by *F. nucleatum*.^33^ Glucose transporter (GLUT) is a key transmembrane protein family essential for cellular glucose transport and metabolism. Intratumoral *F. nucleatum* facilitates the aggregation of GLUT1 on the cell membrane through the N‐acetylgalactosamine (GalNAc)/autophagy/TBC1D5 axis, leading to an increased level of glucose glycolysis and lactate production.^17^ Excessive iron and vitamin K2 exhibit potential anticancer effects. In OPMDs and OSCCs, microbiomes take up extracellular iron and vitamin K2, thereby restricting the utilization by tumor cells. In this way, microbiomes adjust iron and vitamin K2 to suitable levels for cancer progression.^72^ Furthermore, it has been validated in 4‐nitroquinoline‐1‐oxide (4‐NQO)‐induced OSCC models that *P. gingivalis* alters the metabolism of free fatty acids (FFA) in tongue tissues by upregulating fatty acid synthase (FASN) and acetyl‐CoA carboxylase 1 (ACC1) via the de novo fatty acid (FA) synthesis pathway^73^ (Figure 3C).

### 4.4 Activate invasion and metastasis

OSCC originates from the oral epithelium. During carcinogenesis, cell morphology changes, and adhesion between cells and the ECM decreases.^48^

Epithelial cadherin (E‐cadherin) regulates calcium‐dependent cell adhesion in tight junctions, contributing to the cellular structural integrity and stability. E‐cadherin is a well‐established tumor suppressor, particularly in preventing cell invasion and migration.^74^ Studies have demonstrated the downregulation of E‐cadherin in oral epithelial cells and cancer cells infected with bacteria. The reduced E‐cadherin expression disrupts the junction between cells.^75^, ^76^, ^77^, ^78^ In immortalized oral keratinocytes, *P. gingivalis* develops anoikis resistance to sustain invasion. In this process, predominant E‐cadherin expression shifts to neural cadherin (N‐cadherin), which promotes cell detachment and metastasis.^66^ Protein claudin‐4 is also crucial to the formation of tight junction. In OSCC cells, exposure to *Clostridium perfringens'* enterotoxin (CPE) leads to the nuclear translocation of claudin‐4, reducing intercellular adhesion and promoting invasion and migration.^79^ Matrix Metalloproteinases (MMPs) are a family of zinc‐dependent enzymes that degrade components of the ECM, particularly the basement membrane. When cells lose the constraints of the basement membrane, their activity and invasiveness increases.^80^ In OSCC cells infected with *P. gingivalis*, the overexpression of proMMP9 is stimulated through the activation of proteinase‐activated receptor‐4 (PAR‐4) and downstream signaling, including extracellular signal‐regulated kinase 1/2 (ERK1/2), p38, or NF‐κB. Subsequently, the proenzyme is activated in a *P. gingivalis* gingipain proteases‐dependent way to exert the aforementioned functions.^81^, ^82^ Additionally, an upregulation of MMP1, MMP2, MMP7, MMP10 were also observed in cells exposed to bacteria.^78^, ^83^

Epithelial‐mesenchymal transition (EMT) is a reversible transformation in which epithelial cells acquire the traits of mesenchymal cells, including mobility, stemness, and invasiveness.^48^ Long‐term infection by *P. gingivalis* induces EMT phenotype in primary oral epithelial cells (OECs).^78^ In gingival epithelial cells, fimA‐positive *P. gingivalis* activates zinc finger E‐box binding homeobox 1 (ZEB1) and its nuclear translocation, potentially initiating a stable partial EMT process.^84^ Similarly, bacteria promote EMT in OSCC. Studies have reported that periodontal pathogens *P. gingivalis* and *F. nucleatum* upregulate EMT‐related genes, including EGF, TGF‐β1, Twist and SNAI1 in OSCC cells.^62^, ^76^, ^78^, ^85^ *P. gingivalis* and *F. nucleatum* also promote cell invasion and migration via activation of integrin/focal adhesion kinase (FAK) signaling, triggered by TLR/myeloid differentiation primary response 88 (MyD88).^86^ *F. nucleatum* may initiate the EMT through the lncRNA MIR4435‐2HG/miR‐296‐5p/protein kinase B (Akt2)/SNAI1 pathway in cancer cells.^87^ *P. gingivalis* activates JAK1/STAT3 signaling via chemokine (C‐X‐C motif) ligand 2/receptor 2 (CXCL2/CXCR2) axis to promote EMT process.^88^ Furthermore, other signaling pathways such as wingless‐related integration site/nuclear factor of activated T‐cells (Wnt/NFAT) and Notch were also reported to play a role in EMT in OSCC cells infected with bacteria^43^, ^62^, ^88^, ^89^ (Figure 3D).

### 4.5 Induce genome instability and mutation

Microbial genotoxicity induces DNA damage in host cells. As DNA damage intensifies with cell proliferation, the accumulation of gene mutations promotes cancer development.^48^ Periodontal pathogens arrest cell cycle at the S phase, activate upstream signals of ataxia telangiectasia and Rad3‐related protein‐checkpoint kinase 1 (ATR‐CHK1), and inhibit the activation of CHK1, thus triggering DNA damage.^90^ Additionally, study confirmed DNA double‐strand breaks in OSCC cells infected with *F. nucleatum*, mediated by the reduction in Ku70 and P53 expression^56^ (Figure 3E).

### 4.6 Stimulate vasculature

Angiogenesis influences cancer advancement by ensuring a vital nutrient supply to tumors and fostering their continual expansion. Vascular Endothelial Growth Factor (VEGF) is a pivotal signaling protein that regulates angiogenesis. Numerous studies have indicated the production of interleukin‐6 (IL‐6) after the stimulation of bacteria, which subsequently enhances the expression of VEGF in OSCC cells.^52^, ^77^, ^91^ Lipopolysaccharide (LPS) is a major component of the outer membrane of Gram‐negative bacteria, commonly regarded as a potent endotoxin. LPS of *P. gingivalis* promotes the generation of VEGF2 within the gingival epithelial cells, leading to the initiation of blood vessel formation^92^ (Figure 3F).

### 4.7 Promote chronic inflammation

Bacteria promote chronic inflammation directly or indirectly, thereby creating a friendly environment for the progression of OPMDs or OSCC.^93^ Studies have revealed that intralesional microorganisms can activate pro‐inflammatory immune responses both in epithelial cells and OSCC cells, including TLR, STAT, NF‐κB and MAPK signaling pathways.^49^, ^52^, ^62^ OLP is a chronic mucocutaneous inflammatory condition among OPMDs, characterized by a dense T‐cell infiltrate in the lamina propria. Bacteria have been observed to colonize epithelial cells and infiltrate T cells, while the expressions of T cell chemokines CXCL10 and chemokine (C‐C motif) ligand 5 (CCL5) increased. Intracellar bacteria may act as target antigens for T cells to promote basal cell liquefaction and barrier impairment, thus contributing to chronic inflammation.^32^ *Prevotella melaninogenica* promotes IL‐36γ expression via the activation of transient receptor potential vanilloid receptor‐1 (TRPV1) in OLP.^94^ Stimulated by *P. gingivalis* LPS, fibroblasts in OLP lesions undergo transformation into myofibroblasts and secrete pro‐inflammatory cytokines, including IL‐6, IL‐8, and tumor necrosis factor‐alpha (TNF‐α).^95^ Additionally, *P. gingivalis* LPS promotes CCL2 and chemokine (C‐C motif) receptor 2 (CCR2) expressions through TLR‐4/NF‐κB pathway in OLP.^96^ *Fusobacteria* LPS stimulates TLR4 and induces the production of IL‐8 in cancer cells.^62^

**FIGURE 4:**
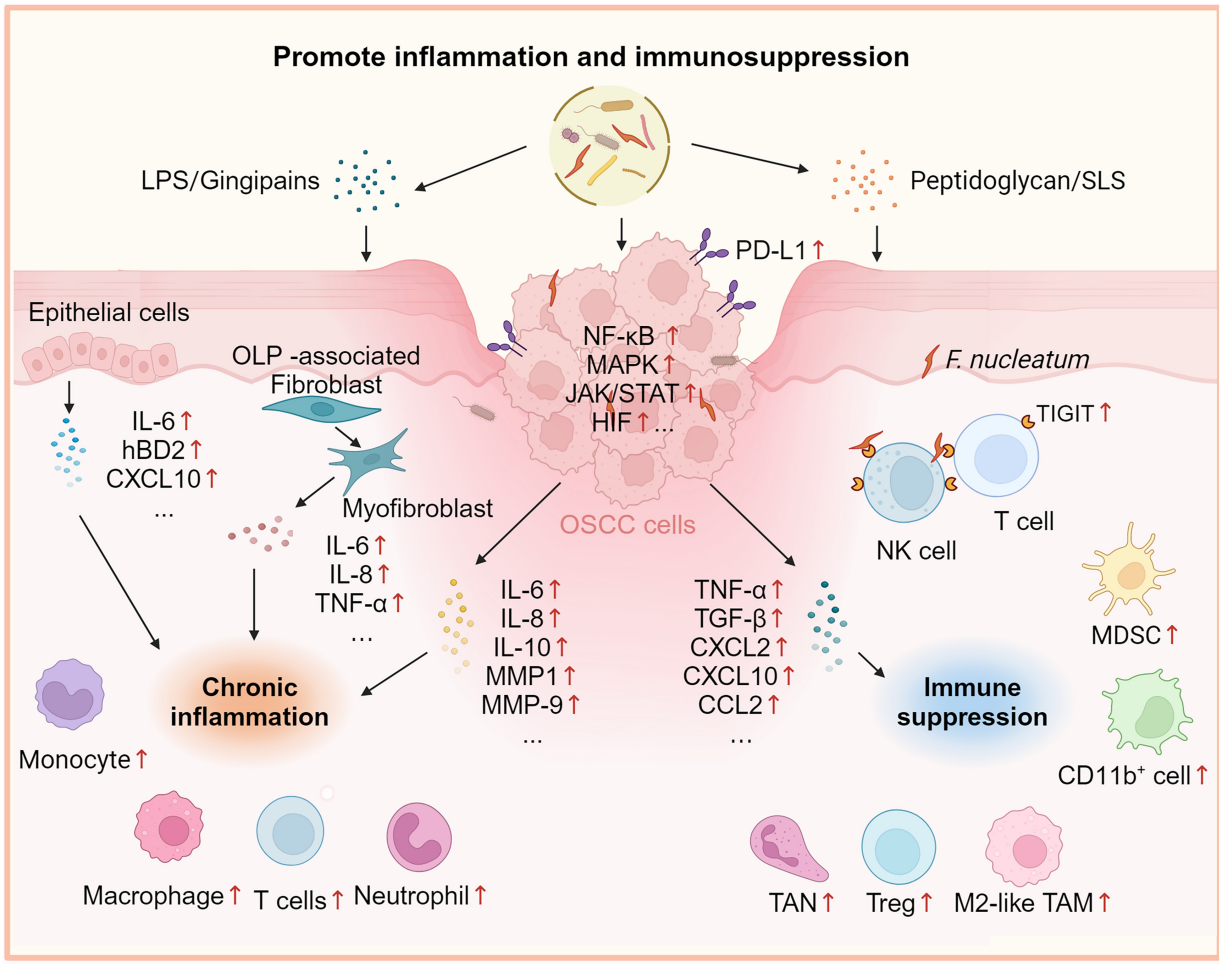
Mechanisms of intralesional bacteria promoting chronic inflammation and immune suppression. Bacteria and their toxins, including streptolysin S (SLS), peptidoglycan, gingipains, and LPS, exert significant influence on the immune microenvironment. They stimulate the secretion of pro‐inflammatory cytokines and leukocyte chemokines by tumor cells and epithelial cells, such as IL‐6, IL‐8, MMPs, and CXCL10. OLP‐associated fibroblasts, when stimulated by LPS, transition into myofibroblasts and release cytokines IL‐6, IL‐8, and TNF‐α. Inflammatory signaling pathways such as NF‐κB, MAPK, JAK/STAT are activated. The chemokines activate and recruit immune cells, including T cells, macrophages, neutrophils, and monocytes, to promote inflammation. Additionally, the level of PD‐L1 in tumor cells increases to evade immune responses. Intralesional *F. nucleatum* inhibits the cytotoxic effect of natural killer (NK) cells and T cells by binding to their T cell immunoglobulin and ITIM domain (TIGIT) receptors through the Fap2 protein. Microbiotas upregulate the expression of TNF‐α, Transforming growth factor‐beta (TGF‐β), CXCL2, CXCL10, and CCL2 in OSCC cells, promoting the infiltration of myeloid‐derived suppressor cells (MDSCs), CD11b + cells, Treg cells, and the formation of tumor‐associated neutrophils (TANs) and M2 polarization of tumor‐associated macrophages (TAMs), which create an immune‐suppressive environment conducive to tumor progression. (Image created with BioRender.com, with permission).

Bacteria also indirectly promote inflammation through the secretion of virulence factors. Gingipains are cysteine proteases produced by *P. gingivalis*, consisting of two types: Arg‐gingipains and Lys‐gingipains. Arg‐gingipains can increase inflammatory cytokine expression in oral keratinocytes through the selective cleavage of PAR‐1.^97^ Streptolysin S (SLS) is a toxin secreted by the β‐hemolytic *S. anginosus* subsp. *anginosus* (β‐SAA). In OSCC cells, SLS facilitates Ca2+ influx, thus increasing the expression of IL‐6 and IL‐8^98^ (Figure 4).

### 4.8 Promote immunosuppression

In oral carcinogenesis, there is an enrichment of immune‐suppressive cells and molecules within the microenvironment.^99^ The TME plays a potential role in creating such a tumor‐friendly condition by selectively enriching the non‐immunogenic or immune‐suppressive bacteria.^28^ A spatial analysis revealed that the distribution of bacteria in OSCC is highly‐organized rather than random. Intratumoral bacterial communities often colonize niches with reduced vascularity and strong immunosuppression. This organization facilitates cancer progression via regulation of immune cells and epithelial cells.^85^

Certain bacteria, including *Rothia* and *Streptococcus*, have a positive correlation with naive and central memory T cells. However, their abundance decreases significantly in the TME, suggesting the diminishing antitumor adaptive immune response in OSCC. Additionally, bacteria such as *Corynebacterium* and *Prevotella* are significantly associated with the expression of GATA3 (a master regulator of T helper 2‐cell differentiation) and IL‐10, which mainly contribute to immune suppression.^28^ Intratumoral *F. nucleatum* can reduce the density of the infiltrated T cells and induce their dysfunction, thereby inhibiting T‐cell immunity.^93^ Moreover, bacteria inhibit immune responses through established inhibitory receptors. Exposure to *Fusobacterium* and *P. gingivalis* upregulates the level of programmed death ligand 1 (PD‐L1). The increased PD‐L1 serves as a mechanism to evade the cytotoxic responses of T cells, potentially via a receptor‐interacting protein kinase 2 (RIP2)‐mediated process.^38^, ^100^ *F. nucleatum* interacts with receptor T cell immunoglobulin and ITIM domain (TIGIT) through Fap2 protein to suppress the immune responses of Natural killer (NK) cells and T cells against OSCC cells.^101^

In addition to directly influencing adaptive immunity, bacteria also influence components of the innate immune system. Myeloid‐derived suppressor cells (MDSCs) exert a potent immunosuppressive ability. In 4‐NQO‐induced OPMDs and OSCC mice, *P. gingivalis* activates the expression of CCL2, CXCL2, IL‐6 and IL‐8 to recruit MDSCs and infiltrate lesions.^102^ M2‐type tumor‐associated macrophages (M2‐TAMs) are a crucial contributor to the inhibition of immune responses in the TME. Periodontal pathogens significantly promote the M2 polarization of macrophages when co‐cultivated with OSCC cells.^103^ In addition, *P. gingivalis* was observed to shield cancer cells from macrophage phagocytosis.^104^ Regarding the underlying mechanism, *P. gingivalis* may facilitate the M2 polarization process through a key modulator downstream of kinase 3 (DOK3) via TNF and MAPK signaling pathways.^105^ In 4‐NQO‐Induced OSCC mice, intratumoral *F. nucleatum* promotes the formation of M2‐TAMs by upregulating lactate expression through GLUT1.^17^ Likewise, tumor‐associated neutrophils‐2 (TANs‐2) exhibit pro‐tumor effects by suppressing immune responses and facilitating proliferation.^106^ Studies have revealed that *P. gingivalis* recruits TANs‐2 via the activation of CXCL2/CXCR2 axis in the TME of OSCC^88^ (Figure 4).

### 4.9 Anti‐cancer effects

Apart from the pro‐cancer effects, some bacteria also play a role in OSCC regression. Periodontitis negative‐associated bacteria (PNB), including *Neisseria sicca* and *Corynebacterium matruchotii*, have the potential to exert anticancer effects. PNB significantly downregulate the level of IL‐6, IL‐8, MMP‐9 and cyclin D1, thereby suppressing the proliferation, migration, and invasiveness of OSCC cells.^107^ In addition, PNB enhance genome stability by activating DNA repair pathways and promoting pyroptosis mediated by the inflammasome containing domains of protein 3 (NLRP3).^108^ *Veillonella parvula* and its major metabolite, sodium propionate (SP), inhibit abnormal proliferation and facilitate apoptosis by blocking the tumor‐associated calcium signal transducer 2 (TROP2)/phosphoinositide 3‐kinase (PI3K)/protein kinase B (Akt) pathway.^109^ *Lactobacillus plantarum* is an important bacterium that commensally lives in the oral cavity. This bacterium induces apoptosis of oral cancer cells through the upregulation of phosphatase and tensin homolog (PTEN) and downregulation of MAPK.^110^ Additionally, multiple studies have reported a higher relative abundance of the genus *Streptococcus* in normal tissues compared to OSCC or OPMDs, suggesting its potential anticancer effect.^28^ The supernatant of *S. anginosus* was observed to promote apoptosis and reduce proliferation and invasion in OSCC cells.^111^ *Streptococcus cristatus* decreases the expression of pro‐inflammatory cytokines by influencing NF‐κB signaling pathways.^112^

**FIGURE 5:**
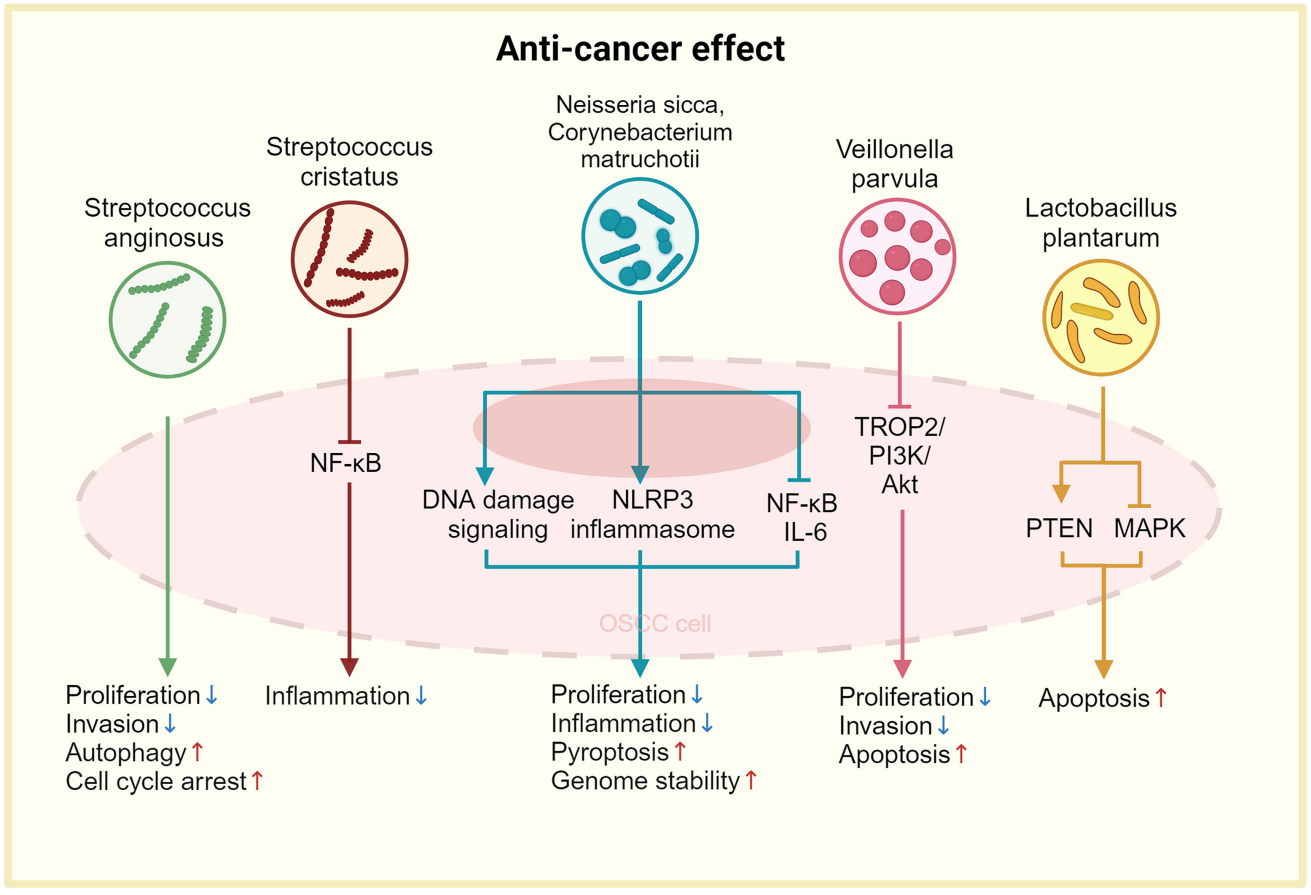
The potential probiotics' anticancer effects. The supernatant of *Streptococcus anginosus* reduces proliferation and invasion and promotes apoptosis in cancer cells. *Streptococcus cristatus* decreases the expression of pro‐inflammatory cytokines by influencing NF‐κB signaling pathways. *Neisseria sicca* and *Corynebacterium matruchotii* significantly inhibit the inflammatory response by down‐regulating NF‐κB and IL‐6 mRNA levels. Moreover, by activating the DNA Damage Response (DDR) and NOD‐like receptor protein 3 (NLRP3) inflammasomes in OSCC cells, they inhibit cell proliferation, promote gene stability, and facilitate pyroptosis (a specific form of programmed cell death mediated by the NLRP3 inflammasome). *Veillonella parvula* and its major metabolite inhibit proliferation, invasion and promote apoptosis by blocking the TROP2/PI3K/Akt pathway. *Lactobacillus plantarum* induces cell apoptosis through the upregulation of PTEN and downregulation of MAPK. (Image created with BioRender.com, with permission).

*P. gingivalis* is relatively special. Despite the majority of studies indicating its tumorigenic effects, there are still researches reporting its anticancer properties and the association with the longer survival in OSCC patients.^46^ A recent study has shown that *P. gingivalis* overturned the immunosuppressive TME via the downregulation of *mucin 1* (*MUC1*).^113^ Phosphoethanolamine dihydroceramide (PEDHC) derived from *P. gingivalis* decreases the level of acid ceramidase and promotes the aggregation of ceramide, thereby accelerating cancer cell death.^114^ Another study reported that *P. gingivalis* induces autophagy and inhibits proliferation of oral cancer cell by downregulating cyclin D1 and cdk4.^115^ These results seem to be contradictory to the other studies. The discrepancy may be attributed to varied status of *P. gingivalis* during cell infection. Heat‐killed *P. gingivalis* shows reduced invasiveness and apoptotic effects compared to live strain.^104^, ^116^ In addition, multiple factors such as cell types, cell status, and duration of treatment also affect the outcomes.^115^ More evidence is needed for a thorough insight of the exact effect of *P. gingivalis* (Figure 5).

## 5 DISCUSSION

OSCC is characterized by high aggressiveness and metastasis, with limited therapeutic options.^117^ Although substantial progress has been achieved in the past decades, patients with advanced‐stage OSCC still face an undesirable prognosis.^118^ This paper provides an overview of the intralesional bacteria in OSCC. Bacteria exhibit varying diversity and composition in different diseases or phases. Particularly, specific bacteria are enriched or reduced at specific stages, such as *Streptococcus*, *Rothia* which are enriched in normal controls but depleted as OSCC progress. This dynamic change provides important clues to the role of bacteria in disease development. The significantly increased bacteria can be potential pathogens that contribute to or adapt to the TME, whereas those declining are more likely part of micro‐ecological balance. Currently, little is known about the exact sources of intralesional microbiota. Understanding of their origins and migration helps identify key bacteria and deepens the comprehension of the microbial mechanisms in diseases.

In terms of the potential roles in OSCC initiation and progression, intralesional bacteria manifest dual nature, with both pro‐carcinogenic and anti‐carcinogenic effects. Representative pro‐carcinogenic bacteria, including *F. nucleatum*, *P. gingivalis* and *T. denticola*, promote the occurrence and development of OSCC through multiple mechanisms, particularly by enhancing proliferation, invasion, inflammation, inhibiting apoptosis, and creating an immunosuppressive microenvironment. On the contrary, anti‐carcinogenic bacteria, notably *N. sicca* and *C. matruchotii*, act as probiotics to prevent the OSCC progression. *P. gingivalis* has been reported to exhibit conflicting pro‐cancer and anti‐cancer effects, which deserves further validation.

Most conclusions rely on 16S rRNA gene sequencing and co‐culture experiments. However, without standardized sampling criteria and laboratory procedures, the comparability of conclusions across different studies may be questionable. In vitro experiments alone cannot entirely replicate the intricate TME and the crosstalk among cells in vivo; co‐culture with individual bacteria also overlooks communications between bacteria; different types of cancer cells may exhibit significant variations in responses to the same bacteria. In addition, the 16S rRNA gene sequencing might encounter challenges when processing samples with low microbial biomass. The limited precision makes it difficult to accurately identify microbial community structures at the species level. Therefore, further research employing new techniques such as 2bRAD is necessary to reveal the biodiversity and variations in gene expression more comprehensively.

Taken together, the intralesional microbiome is a critical component of the pathological microenvironment, which exerts a multifaceted influence on disease progression. There are still many issues to be addressed in this field, requiring technological advancements and concerted efforts. Exploration of intralesional microbiota has considerable clinical significance, which may bring fresh insights into cancer treatment.

## AUTHOR CONTRIBUTIONS

**Zhuoyan Luo:** Data curation (equal); formal analysis (equal); writing – original draft (lead). **Shiping Lv:** Data curation (equal). **Fangzhi Lou:** Investigation (equal). **Li Yan:** Conceptualization (equal); software (equal). **Jingyi Xu:** Formal analysis (equal). **Ning Kang:** Formal analysis (equal). **Yunmei Dong:** Conceptualization (equal); formal analysis (equal); supervision (equal); writing – review and editing (equal). **Xin Jin:** Conceptualization (equal); supervision (equal); writing – review and editing (equal).

## FUNDING INFORMATION

This work was supported by grants from the National Natural Science Foundations of China (No. 82370968) and the Natural Science Foundation of Chongqing (No. CSTB2022NSCQ‐MSX1148). The funding agencies had no role in the study design, collection, analysis, or interpretation of data, writing of the report, or the decision to submit the article for publication.

## CONFLICT OF INTEREST STATEMENT

The authors declare that they have no known competing financial interests or personal relationships that could have appeared to influence the work reported in this paper.

## Data Availability

Data sharing is not applicable to this article as no new data were created or analyzed in this study.
